# Acceptability and feasibility of testing for HIV infection at birth and linkage to care in rural and urban Zambia: a cross-sectional study

**DOI:** 10.1186/s12879-020-4947-6

**Published:** 2020-03-18

**Authors:** Catherine G. Sutcliffe, Jane N. Mutanga, Nkumbula Moyo, Jessica L. Schue, Mutinta Hamahuwa, Philip E. Thuma, William J. Moss

**Affiliations:** 1 Johns Hopkins Bloomberg School of Public Health, 615 N. Wolfe St, Baltimore, MD USA; 2 Livingstone Central Hospital, Livingstone, Zambia; 3 Macha Research Trust, Choma, Zambia

**Keywords:** HIV, Pediatrics, Sub-Saharan Africa, Early infant diagnosis, Zambia, Linkage to care

## Abstract

**Background:**

Early infant diagnosis is important for timely identification of HIV-infected infants and linkage to care. Testing at birth has been implemented to facilitate earlier diagnosis of HIV infection but may present new challenges. This study was conducted to understand the acceptability and feasibility of birth testing in urban and rural settings in southern Zambia.

**Methods:**

This cross-sectional study was conducted at 11 hospitals and clinics in Livingstone, Choma, and Macha in Southern Province, Zambia from 2016 to 2018. Infants born to pregnant women living with HIV at the sites were eligible for enrollment. After enrollment, a questionnaire was administered to the mother and a dried blood spot card was collected from infants for testing at a central laboratory. When results were available, mothers were notified to return to the clinic. Acceptability of birth testing was evaluated based on the proportion of women who agreed to participate and the reasons for non-participation among women who declined. Feasibility of testing at birth was evaluated using turnaround times for returning results, the proportion of women receiving results, and linkage to care for infants testing positive.

**Results:**

One thousand four hundred three women were approached for the study. A small proportion declined due to refusal of birth testing (0 to 8.2% across sites). One thousand two hundred ninety women agreed to have their infants tested. The proportion of mothers receiving results ranged from 51.6 to 92.1%, and was significantly lower at the hospital than clinics in Livingstone (51.6% vs. 69.8%; *p* < 0.0001) and Macha (69.5% vs. 85.7%; p < 0.0001) but not Choma (85.7% vs. 92.1%; *p* = 0.34). For mothers who received test results, the median turnaround time from sample collection was 67 days in Livingstone and 53 days in Macha and Choma. Overall, 23 (1.8%) infants tested positive for HIV but only 8 (34.8%) were linked to care a median of 68 days (range: 29, 784) after sample collection.

**Conclusions:**

While testing at birth was acceptable, this study highlights the operational challenges under a centralized laboratory testing system. Point-of-care platforms are needed for rapid testing and return of results so HIV-infected children can be identified, linked to care, and treated as early as possible.

## Background

In 2018, 1.7 million children were living with HIV, with over 90% residing in sub-Saharan Africa [1]. Despite the scale-up of antiretroviral therapy for pregnant women living with HIV and decreasing rates of mother-to-child transmission, 160,000 children were newly infected with HIV in 2018 [1]. Diagnosing these children and linking them to care and treatment will be key to achieving the 90–90-90 targets for 2020 set by the Joint United Nations Programme on HIV/AIDS [2].

Access to diagnostic testing for infants exposed to HIV has increased over the past decade but remains challenging, particularly in rural settings. Testing commonly occurs in centralized laboratories that are located in urban areas. Clinics within the catchment area collect samples on dried blood spot (DBS) cards that are transported to the laboratory, and results are transported back to the clinic and provided to the mother. Given the number of steps in this process there are many opportunities for delays, such that results are often provided to the mother well beyond the recommended four weeks after sample collection [3] in many areas [4–8]. As a result of these challenges, only 63% of infants in eastern and southern Africa and 21% of infants in western and central Africa [9] are tested as recommended by 8 weeks of age [3].

More recently, testing at birth has been implemented in some settings to identify children infected in utero, address challenges with following up infants at health centers after birth, provide an additional opportunity for testing, and facilitate earlier diagnosis of HIV infection and linkage to care [3, 10], thereby preventing the morbidity and mortality observed in the first few months of life among in utero HIV-infected infants [11]. However, testing around the time of birth and in maternity wards presents challenges that need to be addressed.

This study was conducted to understand the acceptability and feasibility of implementing birth testing at healthcare facilities in both urban and rural settings in southern Zambia.

## Methods

### Study setting

This study was conducted at urban and rural health centers (UHCs and RHCs) and hospitals in three areas in Southern Province, Zambia, including Macha (1 hospital, 4 RHCs), Choma Town (1 hospital, 1 UHC), and Livingstone City (1 hospital, 3 UHCs). Livingstone is a large city near the border with Zimbabwe, with an estimated population of 134,349 in 2010 (Additional File 1) [12]. Prior to 2012, Livingstone was the capital of Southern Province. Choma, now the capital of Southern Province, is a town in the center of Southern Province located halfway between Livingstone and Lusaka, with an estimated population of 51,842 in 2010 [12]. Macha is a rural area located approximately 70 km from Choma Town, with an estimated population density of 25–45 residents per km^2^. The area is primarily populated by subsistence farmers. The HIV prevalence in Southern Province, Zambia was estimated to be 13.3% among adults 15–59 years of age in 2016 [13].

At the time of the study, universal treatment of pregnant women living with HIV was implemented throughout Zambia. Infants exposed to HIV were recommended to be tested at birth (or first contact), 6 weeks, and 6 months of age with a nucleic acid test, and at 9 months, 18–24 months of age or at least 6 weeks post-weaning with a serologic test followed by confirmation with a nucleic acid test if positive [14]. Nucleic acid-based testing was performed at designated central laboratories.

### Study overview and procedures

This analysis was nested within the Novel Screening for Exposed Babies (NSEBA) Study. The objective of the NSEBA Study was to evaluate strategies for implementing point-of-care technologies for early infant diagnosis in Zambia. The NSEBA Study included a cross-sectional validation study of a point-of-care platform for diagnosing HIV at birth conducted at hospitals and clinics in Livingstone, Choma, and Macha from February 2016 to August 2018 (Table 1). This analysis was performed using data collected at birth from the cross-sectional study.

**Table 1:** Description of study sites in Southern Province, Zambia, 2016–2018

Study site	Location	Type of area	Study period	Median (range) HIV+ women delivering per week	Eligible participants
Livingstone Central Hospital	Livingstone City	Urban	Jun 2016 to May 2017 Jun 2017 to Apr 2018	8 (1, 15)	All HIV+ women High-risk HIV+ women^a^
Maramba UHC	Livingstone City	Urban	Jun 2016 to May 2017 Jun 2017 to Apr 2018	3 (0, 12)	All HIV+ women High-risk HIV+ women^a^
Mahatma Gandhi UHC	Livingstone City	Urban	Apr 2017 to Apr 2018	3 (0, 9)	High-risk HIV+ women^a^
Libuyu UHC	Livingstone City	Urban	Apr 2017 to Apr 2018	1 (0, 5)	High-risk HIV+ women^a^
Choma General Hospital	Choma Town	Urban	Aug 2017 to Apr 2018	5 (2, 9)	High-risk HIV+ women^a^
Shampande UHC	Choma Town	Urban	Aug 2017 to Apr 2018	2 (0, 5)	High-risk HIV+ women^a^
Macha Hospital	Macha Area	Rural	Feb 2016 to Aug 2018	2 (0, 9)	All HIV+ women
Mapanza RHC	Macha Area	Rural	Feb 2016 to Aug 2018	0 (0, 4)	All HIV+ women
Moboola RHC	Macha Area	Rural	Feb 2016 to Aug 2018	0 (0, 3)	All HIV+ women
Mangunza RHC	Macha Area	Rural	Feb 2016 to Aug 2018	0 (0, 3)	All HIV+ women
Nalube RHC	Macha Area	Rural	Feb 2016 to Jul 2017	0 (0, 2)	All HIV+ women

*RHC* Rural health center, *UHC* Urban health center

^a^High-risk defined as receiving no antiretroviral drugs throughout pregnancy or starting to receive antiretroviral drugs during pregnancy to prevent mother-to-child transmission

At the Macha sites (1 hospital and 4 RHCs) and for the first year of the study at the Livingstone sites (1 hospital, 2 UHCs), all clinically stable infants born to women living with HIV in the maternity wards were eligible for participation (Table 1). In the second year of the study, additional sites in Choma (1 hospital and 1 UHC) and Livingstone (2 UHCs) were added and eligibility at all sites in Choma and Livingstone was restricted to clinically stable infants born to high-risk women living with HIV to achieve the primary aim of the NSEBA study. High-risk was defined as not receiving antiretroviral drugs throughout pregnancy or starting antiretroviral drugs during pregnancy to prevent mother-to-child transmission of HIV.

Potentially eligible infants were identified by study staff through daily surveillance of admission and delivery registers and their mothers were approached for participation in the study. Mothers who declined participation were asked to provide a reason. Mothers willing to participate were asked to provide written informed consent and then administered a questionnaire to collect information on demographics, antenatal care, HIV testing history, and receipt of antiretroviral drugs. A DBS card was then collected from the infant for testing at a central laboratory (Additional File 2). While national guidelines recommended testing at birth during the study, it was only routinely performed at the central hospital in Livingstone. At all other sites, testing at birth was performed by the study. At sites in Livingstone, DBS cards were sent to the central laboratory in Livingstone. Samples were transported by study staff to the laboratory within 24–48 h of sample collection at hospitals and once per week at clinics, at which time prior available results were retrieved. In Choma and Macha, DBS cards were sent by study staff once per week by courier to a central laboratory in Lusaka. Paper copies of the test results were returned to study staff by courier when available. At all sites, when results were received, study staff contacted the participants by phone and requested they return to the health facility to receive their results. For participants who did not own a mobile phone, communication systems established for routine care (e.g. community lay counselors) were used to contact participants and request they return to the health facility. For infants who tested positive, study staff worked with clinic staff to facilitate and document linkage to care.

### Review of administrative data

An additional study was conducted to provide information on where women living with HIV were delivering and seeking post-natal care. Data were abstracted from the delivery, post-natal, and HIV-exposed registers at the Livingstone and Choma UHCs from 2014 to 2017. At Mahatma Gandhi Clinic in Livingstone and Shampande Clinic in Choma, registers could only be found for 2016 and 2017. All women with documented HIV infection were identified from the registers. Their information was matched between registers based on name, delivery date, and safe motherhood/clinic number. Abstracted data included place and mode of delivery, maternal age, baby’s sex, condition of the baby at delivery, and timing of the first post-natal visit.

### Analysis

The acceptability of testing at birth was evaluated based on the proportion of mothers agreeing to participate in the study and reasons for non-participation among mothers who declined. The feasibility of testing at birth was evaluated using three measures: 1) turnaround times for returning results to the health facility and the mother (reported separately by location of the centralized laboratory); 2) the proportion of mothers who received test results; and 3) linkage to care for infants testing positive. Comparisons were made between hospitals and clinics (UHCs or RHCs) by study location using a Wilcoxon rank sum test for continuous variables and chi-square test for categorical variables. Factors associated with receiving test results were evaluated using chi-square test for categorical variables by study location.

For the administrative data, characteristics of all women and their infants were evaluated by UHC and year. The proportion of women who attended that facility for delivery and post-natal care, only for delivery, or only for post-natal care was calculated separately by clinic and year among mothers who had a live birth and reported being from within the catchment area (< 12 km). As few differences were seen by year (data not shown), data are reported for all years combined by UHC.

All analyses were conducted using SAS, Version 9.4 (SAS Institute Inc., Cary, North Carolina).

### Ethics statement

This study was approved by the Institutional Review Boards at Macha Research Trust and the Johns Hopkins Bloomberg School of Public Health, and by the National Health Research Authority in Zambia. Written informed consent was obtained for participation in the study by trained study staff from a parent or guardian of HIV-exposed infants.

## Results

### Acceptability of testing at birth

During the study period, 1627 pregnant women living with HIV were identified in the maternity wards, including 1403 women with clinically stable infants who were approached for participation in the study (Additional File 3). Most women in all locations (74.2 to 99.5%) agreed to participate and have their infants tested at birth (Table 2). Women who declined were more frequently from the hospitals in Livingstone (5.5%), Choma (10.0%), and Macha (25.8%), where many women preferred not to participate in research (39.3% of declines in Livingstone), preferred not to test at birth (71.4% of declines in Choma), or needed their husband’s permission before participating in the study (39.4% of declines in Macha). Only a small proportion of women approached at each location declined participation because they did not want their infant tested at birth (0 to 8.2%) or preferred testing at the local post-natal clinic (0 to 3.9%). This was particularly true for women delivering at Choma General Hospital (7.1 and 1.4%) and Macha Hospital (8.2 and 3.9%).

**Table 2:** Willingness to enroll and participate in birth testing in Southern Province, Zambia, 2016–2018

	Livingstone City		Choma Town		Macha Area	
	Hospital^d^	Urban Health Centers	Hospital	Urban Health Center	Hospital	Rural Health Centers
Number of pregnant women living with HIV approached to participate in the study	513	315	70	39	256	210
Number of women agreed to participate^a^	485 (94.5)	305 (96.8)	63 (90.0)	38 (97.4)	190 (74.2)	209 (99.5)
Number of women declined^a^	28 (5.5)	10 (3.2)	7 (10.0)	1 (2.6)	66 (25.8)	1 (0.5)
Reasons declined^b^,^c^						
Doesn’t want child tested at birth	10 (35.7/1.9)	4 (40.0/1.3)	5 (71.4/7.1)	1 (100.0/2.6)	21 (31.8/8.2)	0
Prefers child to be tested at post-natal clinic	0	0	1 (14.3/1.4)	0	10 (15.2/3.9)	0
Prefers not to participate in research	11 (39.3/2.1)	4 (40.0/1.3)	1 (14.3/1.4)	0	5 (7.6/2.0)	1 (100.0/.5)
Prefers not to have extra blood drawn	0	2 (20.0/0.6)	0	0	0	0
Needs to ask husband’s permission/Husband refused	4 (14.3/0.8)	1 (10.0/0.3)	0	0	26 (39.4/10.2)	0
Mother in a hurry to get home	0	0	0	0	5 (7.6/2.0)	0
Other/unknown	3 (10.7/0.6)	1 (10.0/0.3)	0	0	1 (1.5/0.4)	0

^a^ Numbers represent n (% among women approached)

^b^ Multiple reasons could be specified

^c^ Numbers represent n (% among women who declined / among all women approached)

^d^ Birth testing was a routine procedure at the hospital in Livingstone; at all other sites, birth testing was performed as a study procedure

### Feasibility of testing at birth

Testing at birth was performed for 1290 infants exposed to HIV. The characteristics of the mothers and infants differed by site (Table 3): mothers and fathers enrolled in the Macha sites were less educated; mothers enrolled in the Choma sites were less likely to have received PMTCT as enrollment was restricted to high-risk women living with HIV; mothers enrolled from the hospitals at all sites and from the UHCs in Livingstone were more likely to intend to take the infant to a different facility for post-natal care; and mothers enrolled in Macha and from the UHCs in Choma were less likely to have access to a cell phone.

**Table 3:** Characteristics of study participants in Southern Province, Zambia, 2016–2018

	Livingstone City		Choma Town		Macha Area		***p***-value
	Hospital (***n*** = 485)	Urban Health Centers (***n*** = 305)	Hospital (***n*** = 63)	Urban Health Center (***n*** = 38)	Hospital (***n*** = 190)	Rural Health Centers (***n*** = 209)	
Mother’s age in years – median (IQR)	30 (24, 34)	28 (24, 32)	26 (22, 33)	27 (23, 33)	32 (23, 37)	29 (24, 35)	0.0006
Mother’s education, n (%)							< 0.0001
None/primary	105 (21.7)	77 (25.3)	21 (33.3)	16 (42.1)	110 (57.9)	119 (56.9)	
Secondary	309 (63.7)	208 (68.2)	39 (61.9)	19 (50.0)	68 (35.8)	85 (40.7)	
More than high school	70 (14.4)	18 (5.9)	3 (4.8)	1 (2.6)	7 (3.7)	4 (1.9)	
Unknown	1 (0.2)	2 (0.7)	0	2 (5.3)	5 (2.6)	1 (0.5)	
Father’s education, n (%)							< 0.0001
None/primary	16 (3.3)	16 (5.3)	14 (22.2)	3 (7.9)	62 (32.6)	68 (32.5)	
Secondary	294 (60.6)	208 (68.2)	39 (61.9)	26 (68.4)	73 (38.4)	102 (48.8)	
More than high school	111 (22.9)	49 (16.1)	5 (7.9)	5 (13.2)	13 (6.8)	8 (3.8)	
Unknown	64 (13.2)	32 (10.5)	5 (7.9)	4 (10.5)	42 (22.1)	31 (14.8)	
Mother received drugs for PMTCT, n (%)	445 (91.8)	265 (86.9)	46 (73.0)	29 (76.3)	169 (89.0)	195 (93.3)	< 0.0001
Child’s age in hours – median (IQR)	11 (5, 21)	5 (2, 10)	20 (12, 68)	5 (3, 10)	17 (9, 25)	10 (5, 15)	< 0.0001
Child sex – female, n (%)	230 (47.4)	164 (53.8)	29 (46.0)	13 (34.2)	109 (57.4)	107 (51.2)	0.05
Returning to study location for post-natal care, n (%)	22 (4.5)	168 (55.1)	2 (3.2)	35 (92.1)	74 (39.0)	187 (89.5)	< 0.0001
Mother has access to a cell phone, n (%)	373 (76.9)	234 (76.7)	48 (76.2)	26 (68.4)	115 (60.5)	135 (64.9)	< 0.0001

**Fig. 1:**
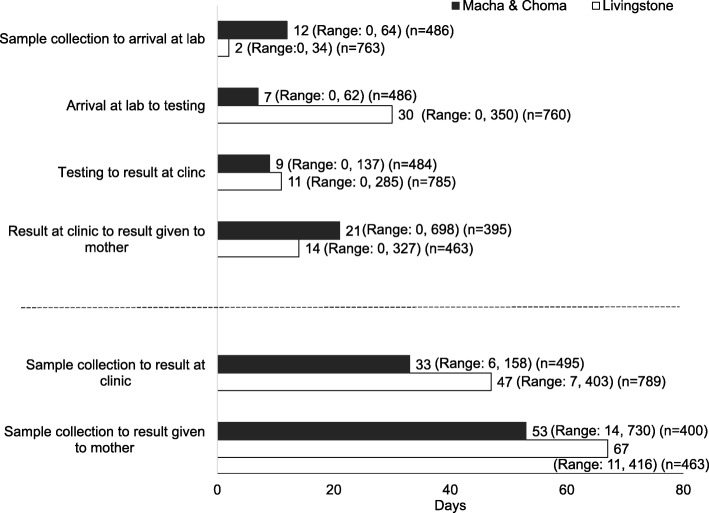
Median turnaround time (in days) for test results by study location

The turnaround times for each step in the testing process were documented and are shown in Fig. 1 (Additional File 4). There were significant differences in the turnaround times between the Livingstone, Macha, and Choma sites for each step. Due to the use of a courier system and the distances traveled by participants in the Macha area, sites in Macha and Choma had significantly longer median times from sample collection to arrival at the laboratory compared to Livingstone (12 vs. 2 days; *p* < 0.0001) and from the result arriving at the clinic to being given to the mother (21 vs. 14 days; p < 0.0001). However, sites in Livingstone experienced a significantly longer median time from arriving at the laboratory to testing compared to Macha and Choma (30 vs. 7 days; *p* < 0.0001). Consequently, sites in Livingstone had a significantly longer median turnaround time from sample collection to the result returning to the clinic (47 vs. 33 days; *p* < 0.0001) and from sample collection to the result being given to the mother (67 vs. 53 days; *p* < 0.004), despite closer proximity to the central laboratory. Changes to testing capacity at the central laboratory, including reagent stockouts and procurement of additional PCR instruments, also impacted turnaround times in Livingstone (Additional File 5).

**Fig. 2:**
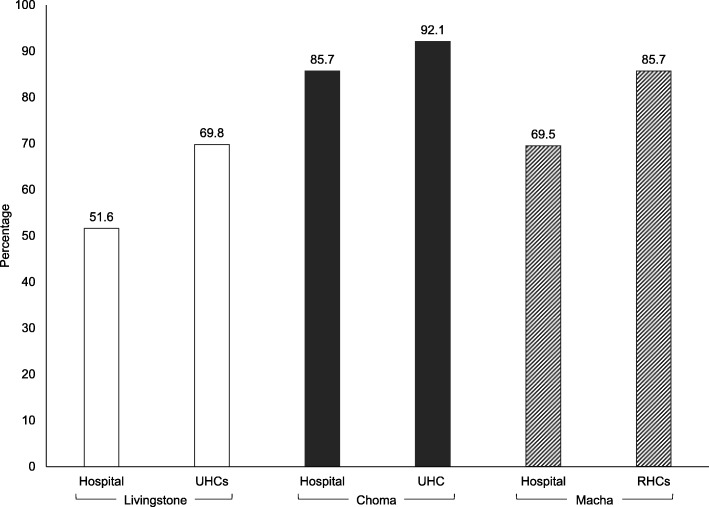
Proportion of HIV DNA test results returned to mothers by study location. RHC: rural health center; UHC: urban health center

The proportion of mothers receiving results ranged from 51.6% at the hospital in Livingstone to 92.1% at the UHC in Choma (Fig. 2), and was significantly lower at the hospitals than the UHCs in Livingstone (51.6% vs. 69.8%; *p* < 0.0001) and RHCs in Macha (69.5% vs. 85.7%; p < 0.0001) but not Choma (85.7% vs. 92.1%; *p* = 0.34). There was significant variation in the proportion of mothers receiving results at the UHCs in Livingstone (46.3 to 75.6%; p < 0.0001) but not in Macha (82.3 to 100.0%; *p* = 0.28) (Additional File 6). Common reasons for not receiving results were that mothers did not return for test results (39.3%; mother alerted to availability of results by phone but did not show up), mothers could not be reached by phone (54.8%; mothers either had no phone, were not reachable, or gave an incorrect or non-working number), or mothers moved or lived out of the study area (2.8%). The primary reasons varied by study location (Additional File 7). Factors associated with receiving test results varied by study location (Additional File 8). In Livingstone, being seen in a UHC, returning to the same clinic for post-natal care, having access to a cell phone, and higher maternal and paternal education were associated with a higher likelihood of receiving test results. In Choma, only higher paternal education was associated with a higher likelihood of receiving test results. In Macha, being seen in a RHC, returning to the same clinic for post-natal care, shorter travel time, and being at low risk for mother-to-child transmission were associated with a higher likelihood of receiving test results.

### Linkage to care

Overall, 23 (1.8%) infants tested positive for HIV, including 10 (2.1%) at the hospital in Livingstone, 8 (2.6%) at the Livingstone UHCs, 2 (3.2%) at the hospital in Choma, none at the Choma UHC, 2 (1.1%) at the hospital in Macha, and 1 (0.5%) at the Macha RHCs (an additional 3 tests were invalid, 1 test was indeterminate, and 1 test result was lost). Only 8 (34.8%) infants were linked to care and started on ART. The median time from the mother receiving results to ART initiation was 10 days (IQR: 0, 68; range: 0, 715), but the median time from sample collection to ART initiation was 68 days (IQR: 34, 173; range: 29, 784; Additional File 9). For the 15 infants not linked to care, reasons included the mother not receiving the results (46.7%; *n* = 7), refusing treatment (20.0%; *n* = 3), living in another city (20.0%; n = 3), the child died (6.7%; *n* = 1), and the parents preferring to wait for confirmatory testing at their local clinic (6.7%; n = 1).

### Administrative data

**Fig. 3:**
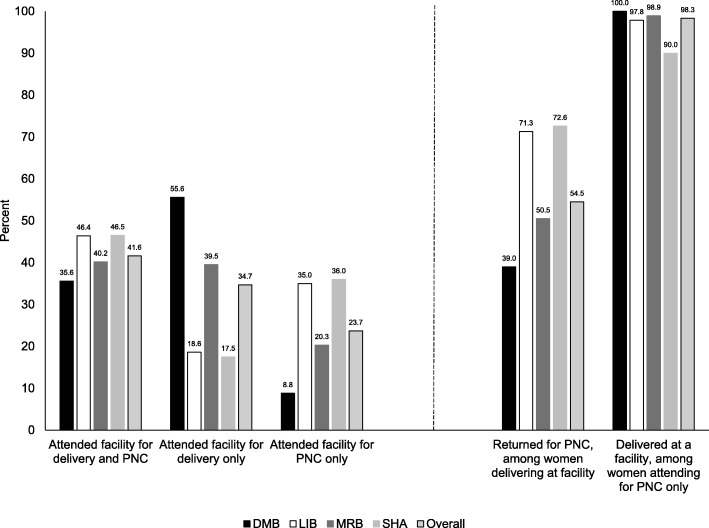
Attendance at facilities for delivery and post-natal care in Livingstone, 2014–2017. DMB: Mahatma Gandhi Urban Health Center; LIB: Libuyu Urban Health Center; MRB: Maramba Urban Health Center; PNC: post-natal care; SHA: Shampande Urban Health Center

The characteristics of the 2078 women living with HIV and their infants attending each facility for either delivery or post-natal care are presented in Additional File 10. Among the 1821 women who had a live birth and reported residing within the catchment area of the facility, attendance at the facility for delivery and post-natal care was evaluated. Less than half of the women attended the facility for both delivery and post-natal care (35.6–46.5%; Fig. 3). Among women delivering at the facility, there was significant variation across clinics in the proportion returning for post-natal care (39.0–72.6%) Among women attending a facility just for post-natal care, most (90.0–100.0%) delivered at another health facility.

## Discussion

In this study in southern Zambia, birth testing for early infant diagnosis under the current centralized testing system was challenging. While testing at birth in both urban and rural areas was acceptable to most women, many did not return for the results, such that only a third of infants diagnosed with HIV were linked to care. Turnaround times for results from the central laboratory were long for both rural and urban clinics, adding to the challenges of early infant diagnosis.

Prior to implementation and release of recommendations around birth testing, concerns were raised about the acceptability of testing at birth. In a qualitative study among women living with HIV in Namibia, Kenya, and Nigeria who had not undergone birth testing, participants were concerned that finding out the child’s HIV infection status so soon after delivery would have a negative psychological impact on the mother [15]. In addition, they raised fears about disclosure of their HIV status to their partner or family if testing was done in maternity wards [15]. In this study, most women agreed to have their infants tested, adding to the literature suggesting that testing at birth is acceptable. Other studies evaluating birth testing after implementation, including in Lesotho [16] and South Africa [17], also found high acceptance. In Lesotho, the primary reported benefit was earlier knowledge of the child’s status, which facilitated better care and relieved the caregiver’s anxiety over the child’s status [5, 16]. While refusals were rare, they did occur, primarily due to fear of hurting the newborn by drawing blood, long waiting times for blood draws, and fear of finding out the child was infected with HIV [16]. In this study, underlying reasons for refusing testing at birth were not ascertained. However, some women declined participation because they preferred testing at their post-natal clinic. This occurred more often at the hospitals than clinics where women were more likely to seek post-natal care at another facility.

Care seeking at different facilities for delivery and post-natal care presented a major challenge for implementation of birth testing in both urban and rural settings in this study, particularly at the hospitals and in Livingstone. As a result, staff had to contact women when results were available, which was challenging in the context of limited access to phones and cellular connectivity, leading to up to half of participants not receiving test results in Livingstone. These challenges were also found through review of clinic registers, and were described in the pilot study in Lesotho [16].

Health seeking behaviors around birth and the post-natal period were challenging for birth testing due to the lengthy and variable turnaround time for results from the central laboratories. The median turnaround times from sample collection to return of results to the clinic were 33 days for the Macha and Choma sites and 47 days for the Livingstone sites, similar to turnaround times observed in Lesotho [5]. However, the range of turnaround times was as short as 6 days and as long as 403 days, making it difficult to advise women on when to return to the facility. Testing at sites in Livingstone represents the testing experience for clinics located in the same area as the central laboratory. While turnaround times in Livingstone were consistent with another study from this site [18], they were longer than expected during the study due to two documented reagent stock-outs. This led to pauses in testing while reagents were procured and additional delays due to a backlog of samples. Testing at sites in Macha and Choma represents the testing experience for clinics located far from the central laboratory. However, as testing at the central laboratory in Lusaka was performed as a study procedure and the process was managed by study staff, the turnaround times for this study were significantly shorter than those observed under routine conditions in this area [19–21]. In all study areas, the time to get results to the mother was longer than the 4 weeks recommended by WHO [3]. Contacting participants when results were available was also challenging, as up to 40% of women did not have access to a cell phone and unreliable network coverage limited access for women who did.

As a result of the challenges observed, only a third of HIV-infected infants were linked to care. This finding supports the need for point-of-care testing in this setting, particularly in hospitals and urban facilities, so that results can be returned before mothers leave the facility. Several point-of-care platforms, including the m-PIMA (Abbott Laboratories, Chicago, Illinois) and GeneXpert (Cepheid Inc., Sunnyvale, California), are available and have been validated for testing at birth [22, 23]. In South Africa, use of a point-of-care test at birth increased the proportion of mothers receiving results from 53 to 96% [23]. In addition, randomized trials of point-of-care testing for early infant diagnosis found a significant decrease in the time to treatment initiation for HIV-infected infants with use of these platforms [24, 25]. While point-of-care platforms may improve testing, new operational challenges may emerge at the facilities that need to be addressed, as observed with point-of-care CD4 testing [26]. Alternatively, given that testing at birth only identifies infants infected in utero and the low rate of HIV transmission observed with high PMTCT coverage, the utility of investing further resources into testing at birth should be considered. A cost-effectiveness analysis comparing birth and 6 week testing in a centralized system found that strengthening testing at 6 weeks was more cost-effective than adding testing at birth [27].

This study had several limitations. First, testing at birth was evaluated in the context of a study and so acceptability may have been underestimated. Once testing is routinely recommended, fewer women may decline. In addition, follow-up to provide results was performed by dedicated study staff, which may have led to an overestimation of results being returned and linkage to care. Second, this study was facility-based and so women delivering at home were not included. In 2018, an estimated 82.1% of women in Southern Province delivered at a health facility [28]. These women may bring their infants for testing early in the post-natal period, which was not evaluated in this study. Third, the analysis involving administrative data was limited by the quality of the registers. Many records were missing data, thus limiting linkage between registers. Consequently, the proportion of women delivering and receiving post-natal care at a clinic may have been underestimated. Lastly, this study was conducted in selected clinics in three areas of Southern Province and therefore results may not be generalizable to other areas in Zambia or the region. However, while the estimates for each outcome may differ between clinics and regions, the challenges identified are likely to be similar.

## Conclusions

HIV testing at birth in maternity wards was found to be acceptable to most women. However, long turnaround times for results and the use of multiple health facilities for delivery and post-natal care resulted in up to half of women failing to receive test results and only a third of HIV-infected infants being linked to care. This study highlights the challenges of implementing birth testing under a centralized testing system and the need for point-of-care platforms for rapid testing and return of results so that HIV-infected children can be identified and treated as early as possible.

## Supplementary information

**Additional file 1 **Map of study areas and sites in Southern Province, Zambia. Map of Southern Province, Zambia. *Google Maps,* September 2019 (https://www.google.com/maps/place/Southern+Province,+Zambia/@-16.68371,25.8207546,8z/data=!3m1!4b1!4m5!3m4!1s0x1946958bdd263cdd:0xbff302af89f265b4!8m2!3d-16.9620634!4d26.419389)

**Additional file 2.** Study procedures for hospitals and clinics in southern Zambia, 2016–2018

**Additional file 3.** Study flow chart. RHC: rural health center; UHC: urban health center

**Additional file 4.** Distribution of turnaround times from sample collection to results returned to the clinics (A) and to the mother (B) in southern Zambia, 2016–2018

**Additional file 5.** Median turnaround time (in days) for test results in Livingstone by operational period. Period 1: sample collected June 30, 2016 (study start) to September 30, 2016 – normal lab operation with one PCR instrument; Period 2: sample collected October 1, 2016 to December 13, 2016 – reagent stockout; Period 3: sample collected December 14, 2016 to August 17, 2017 – normal lab operation with one PCR instrument; Period 4: sample collected August 18, 2017 to October 30, 2017 – normal lab operation with two PCR instruments; Period 5: sample collected October 31, 2017 to December 14, 2017 – reagent stockout; Period 6: sample collected December 15, 2017 to April 10, 2018 (study end) – normal lab operation with two PCR instruments.

**Additional file 6.** Proportion of HIV test results returned to mothers by study location. LCH: Livingstone Central Hospital; MRB: Maramba Urban Health Center; DMB: Mahatma Gandhi Urban Health Center; LIB: Libuyu Urban Health Center; CGH: Choma General Hospital; SHA: Shampande Urban Health Center; MMH: Macha Hospital; MPZ: Mapanza Rural Health Center; MOB: Moobola Rural Health Center; MGZ: Mangunza Rural Health Center; NLB: Nalube Rural Health Center.

**Additional file 7.** Reasons that HIV test results were not returned to mothers by study location

**Additional file 8.** Factors associated with receiving test results for early infant diagnosis by study location

**Additional file 9.** Timing of testing and ART initiation for HIV-infected infants linked to care

**Additional file 10.** Characteristics of women living with HIV and their infants attending study sites for delivery and post-natal care, 2014–2017

## Data Availability

Under the Research Health Act, the Government of Zambia does not allow public access to data collected in Zambia. All investigators interested in the data are required to submit a written request to the Ministry of Health. Contact Dr. Catherine Sutcliffe (csutcli1@jhu.edu) to coordinate the request.

## Abbreviations

ART: Antiretroviral therapy
HIV: Human immunodeficiency virus
AIDS: Acquired immunodeficiency syndrome
PMTCT: Prevention of mother to child transmission of HIV
UHC: Urban health center
RHC: Rural health center
