# Supplementary figures and images for: Acceptability and feasibility of testing for HIV infection at birth and linkage to care in rural and urban Zambia: a cross-sectional study
Source: BMC Infect Dis. 2020 Mar 18;20:227. doi: 10.1186/s12879-020-4947-6 (PMC7079396; doi:10.1186/s12879-020-4947-6)

## Slide 1
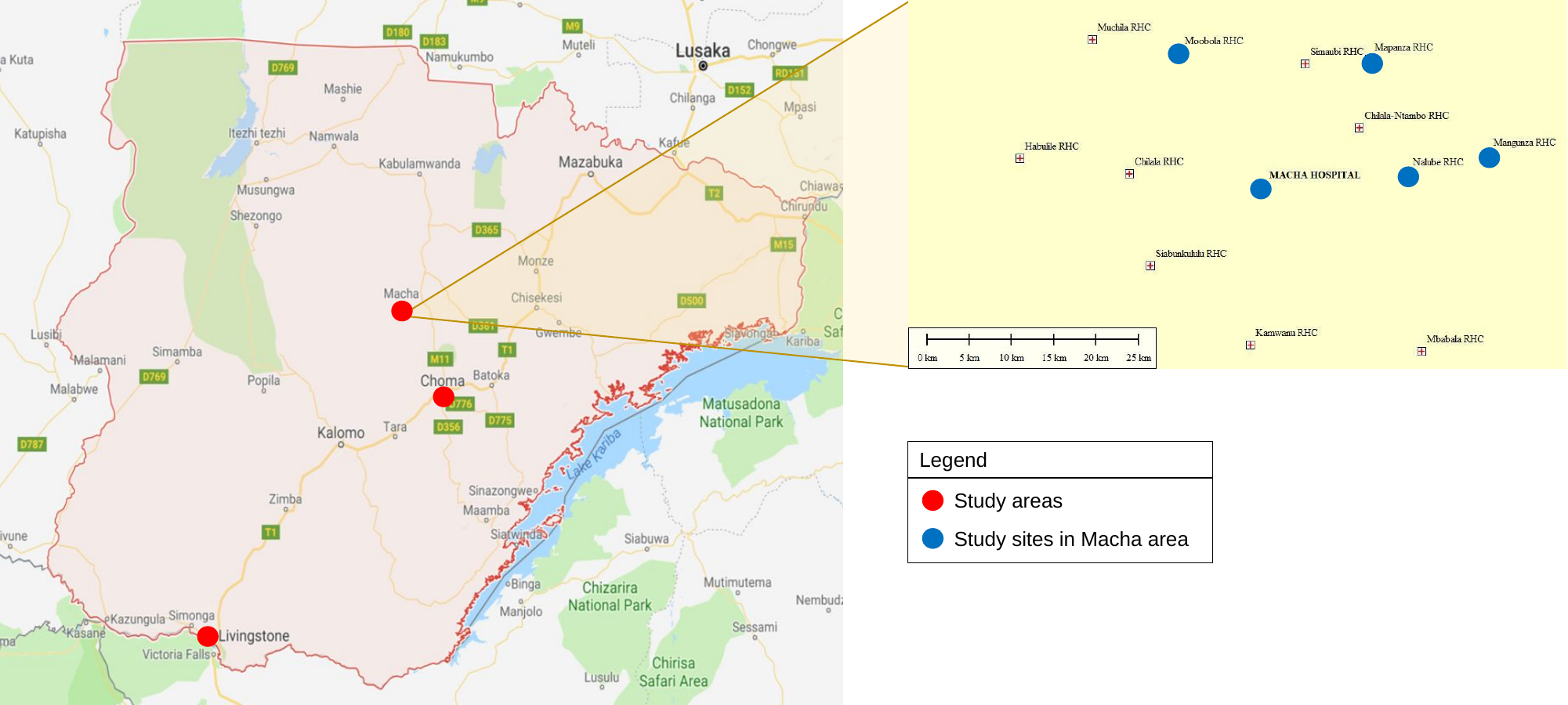

Legend
Study areas
Study sites in Macha area

Supplement: Supplementary file 1 — Additional file 1 Map of study areas and sites in Southern Province, Zambia. Map of Southern Province, Zambia. Google Maps, September 2019 (https://www.google.com/maps/place/Southern+Province,+Zambia/@-16.68371,25.8207546,8z/data=!3m1!4b1!4m5!3m4!1s0x1946958bdd263cdd:0xbff302af89f265b4!8m2!3d-16.9620634!4d26.419389) [file 12879_2020_4947_MOESM1_ESM.pptx]

## Slide 1
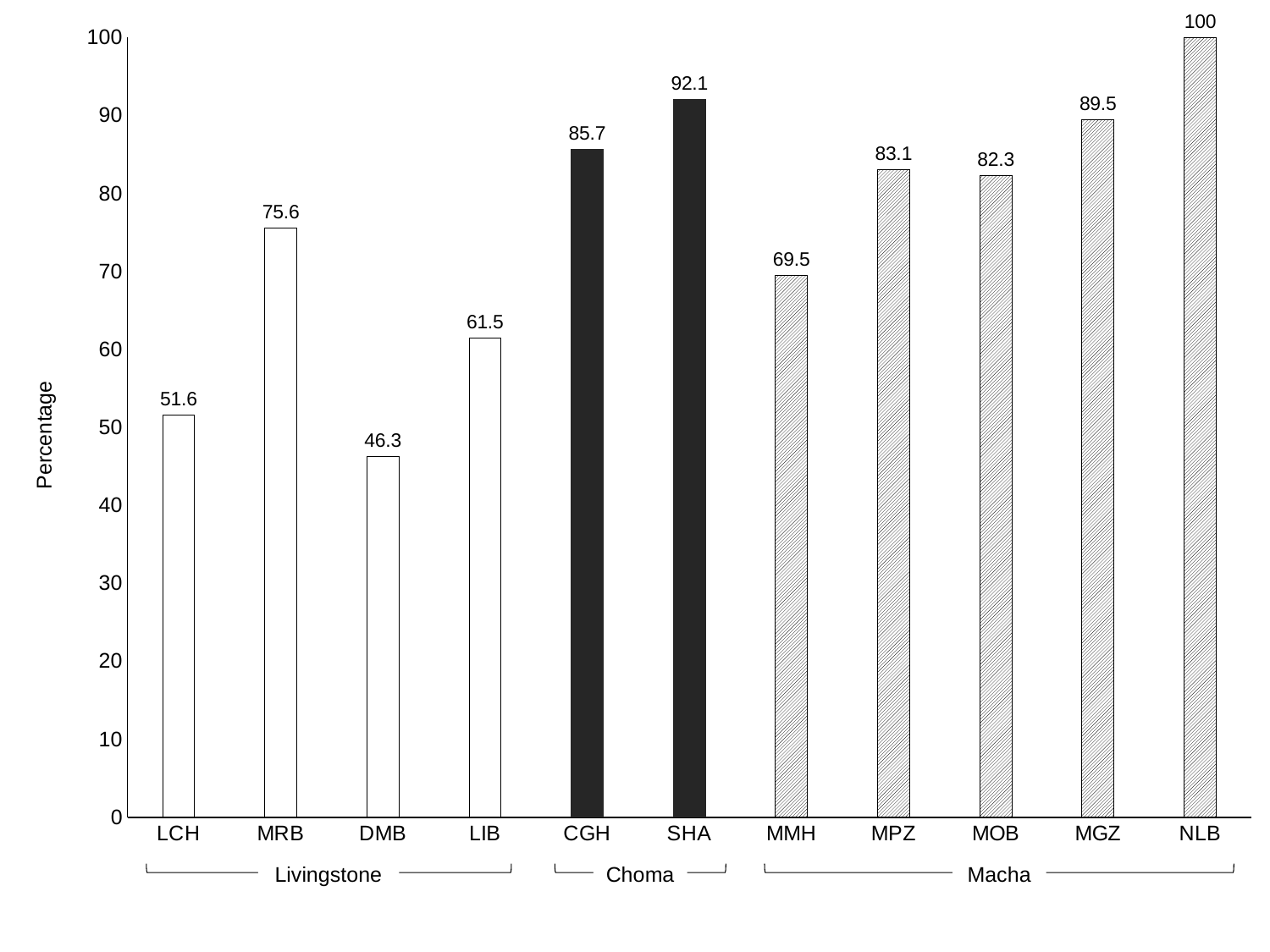

### Chart
| Category | Series 1 |
|---|---|
| LCH | 51.6 |
| MRB | 75.6 |
| DMB | 46.3 |
| LIB | 61.5 |
| CGH | 85.7 |
| SHA | 92.1 |
| MMH | 69.5 |
| MPZ | 83.1 |
| MOB | 82.3 |
| MGZ | 89.5 |
| NLB | 100.0 |
Livingstone
Choma
Macha

Supplement: Supplementary file 6 — Additional file 6. Proportion of HIV test results returned to mothers by study location. LCH: Livingstone Central Hospital; MRB: Maramba Urban Health Center; DMB: Mahatma Gandhi Urban Health Center; LIB: Libuyu Urban Health Center; CGH: Choma General Hospital; SHA: Shampande Urban Health Center; MMH: Macha Hospital; MPZ: Mapanza Rural Health Center; MOB: Moobola Rural Health Center; MGZ: Mangunza Rural Health Center; NLB: Nalube Rural Health Center. [file 12879_2020_4947_MOESM6_ESM.pptx]
