# Supplementary material for: Acceptability and feasibility of testing for HIV infection at birth and linkage to care in rural and urban Zambia: a cross-sectional study
Source: BMC Infect Dis. 2020 Mar 18;20:227. doi: 10.1186/s12879-020-4947-6 (PMC7079396; doi:10.1186/s12879-020-4947-6)
Supplement: Supplementary file 2 — Additional file 2. Study procedures for hospitals and clinics in southern Zambia, 2016–2018 [file 12879_2020_4947_MOESM2_ESM.pptx]

## Slide 1
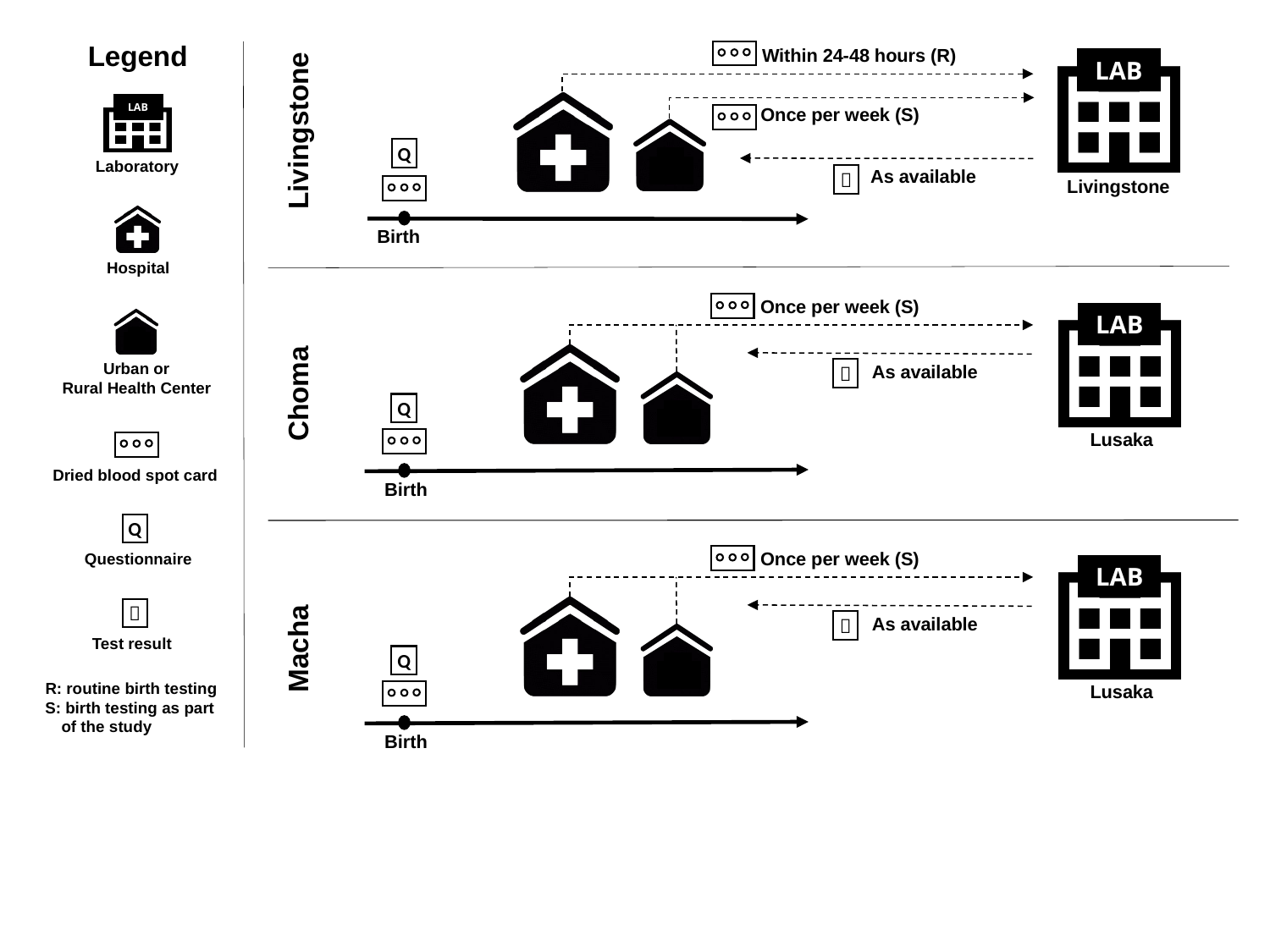

Legend
Within 24-48 hours (R)
LAB
LAB
Once per week (S)
Livingstone
Q
Laboratory
As available

Livingstone
Birth
Hospital
Once per week (S)
LAB
Urban or
Rural Health Center
As available

Choma
Q
Lusaka
Dried blood spot card
Birth
Q
Once per week (S)
Questionnaire
LAB

As available

Macha
Test result
Q
R: routine birth testing
S: birth testing as part of the study
Lusaka
Birth
