# Supplementary material for: Acceptability and feasibility of testing for HIV infection at birth and linkage to care in rural and urban Zambia: a cross-sectional study
Source: BMC Infect Dis. 2020 Mar 18;20:227. doi: 10.1186/s12879-020-4947-6 (PMC7079396; doi:10.1186/s12879-020-4947-6)
Supplement: Supplementary file 3 — Additional file 3. Study flow chart. RHC: rural health center; UHC: urban health center [file 12879_2020_4947_MOESM3_ESM.pptx]

## Slide 1
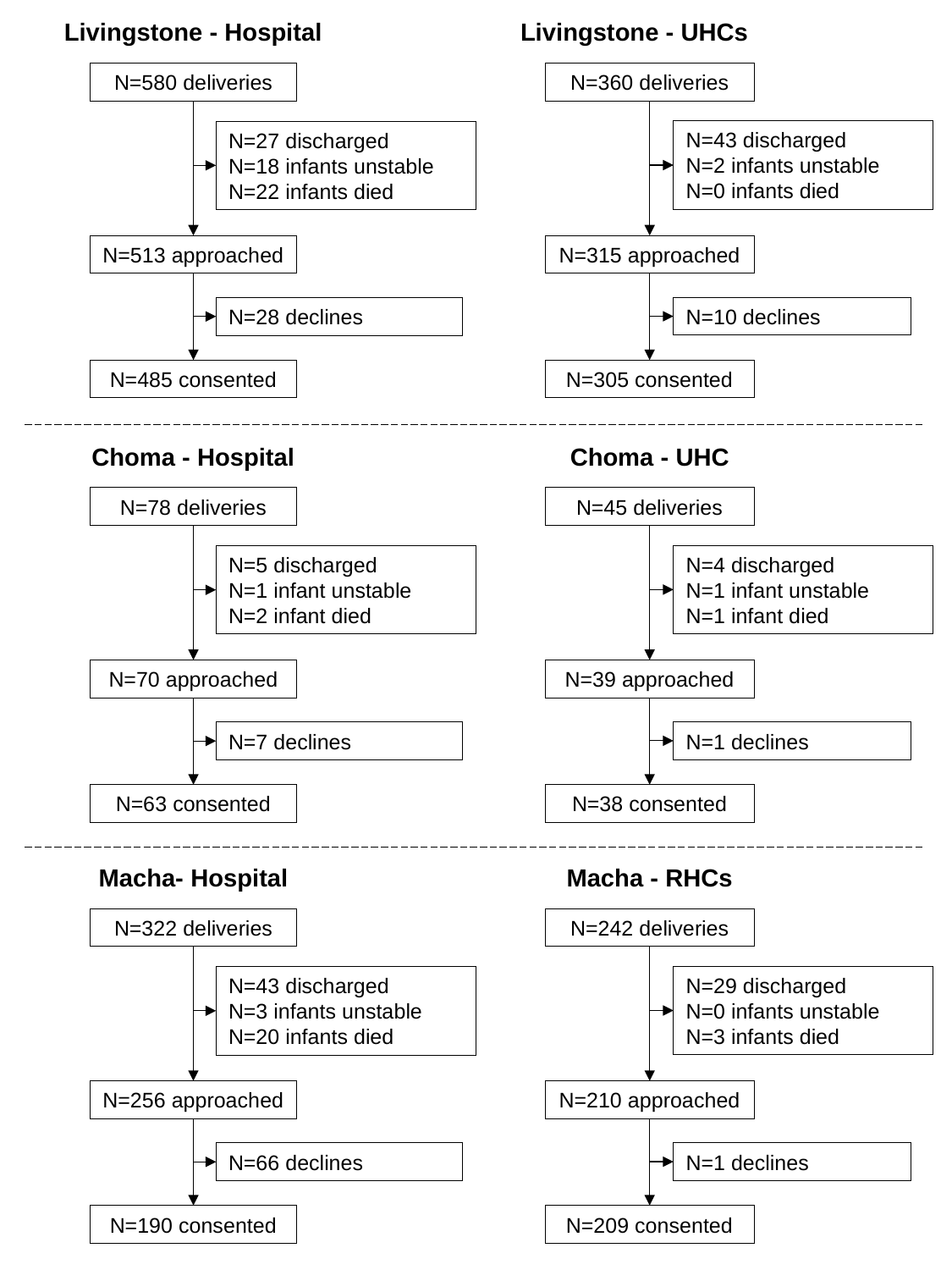

Livingstone - Hospital
Livingstone - UHCs
N=580 deliveries
N=360 deliveries
N=43 discharged
N=2 infants unstable
N=0 infants died
N=27 discharged
N=18 infants unstable
N=22 infants died
N=513 approached
N=315 approached
N=10 declines
N=28 declines
N=485 consented
N=305 consented
Choma - Hospital
Choma - UHC
N=78 deliveries
N=45 deliveries
N=4 discharged
N=1 infant unstable
N=1 infant died
N=5 discharged
N=1 infant unstable
N=2 infant died
N=70 approached
N=39 approached
N=1 declines
N=7 declines
N=63 consented
N=38 consented
Macha- Hospital
Macha - RHCs
N=322 deliveries
N=242 deliveries
N=29 discharged
N=0 infants unstable
N=3 infants died
N=43 discharged
N=3 infants unstable
N=20 infants died
N=256 approached
N=210 approached
N=1 declines
N=66 declines
N=190 consented
N=209 consented
