# Supplementary material for: Acceptability and feasibility of testing for HIV infection at birth and linkage to care in rural and urban Zambia: a cross-sectional study
Source: BMC Infect Dis. 2020 Mar 18;20:227. doi: 10.1186/s12879-020-4947-6 (PMC7079396; doi:10.1186/s12879-020-4947-6)
Supplement: Supplementary file 4 — Additional file 4. Distribution of turnaround times from sample collection to results returned to the clinics (A) and to the mother (B) in southern Zambia, 2016–2018 [file 12879_2020_4947_MOESM4_ESM.pptx]

## Slide 1
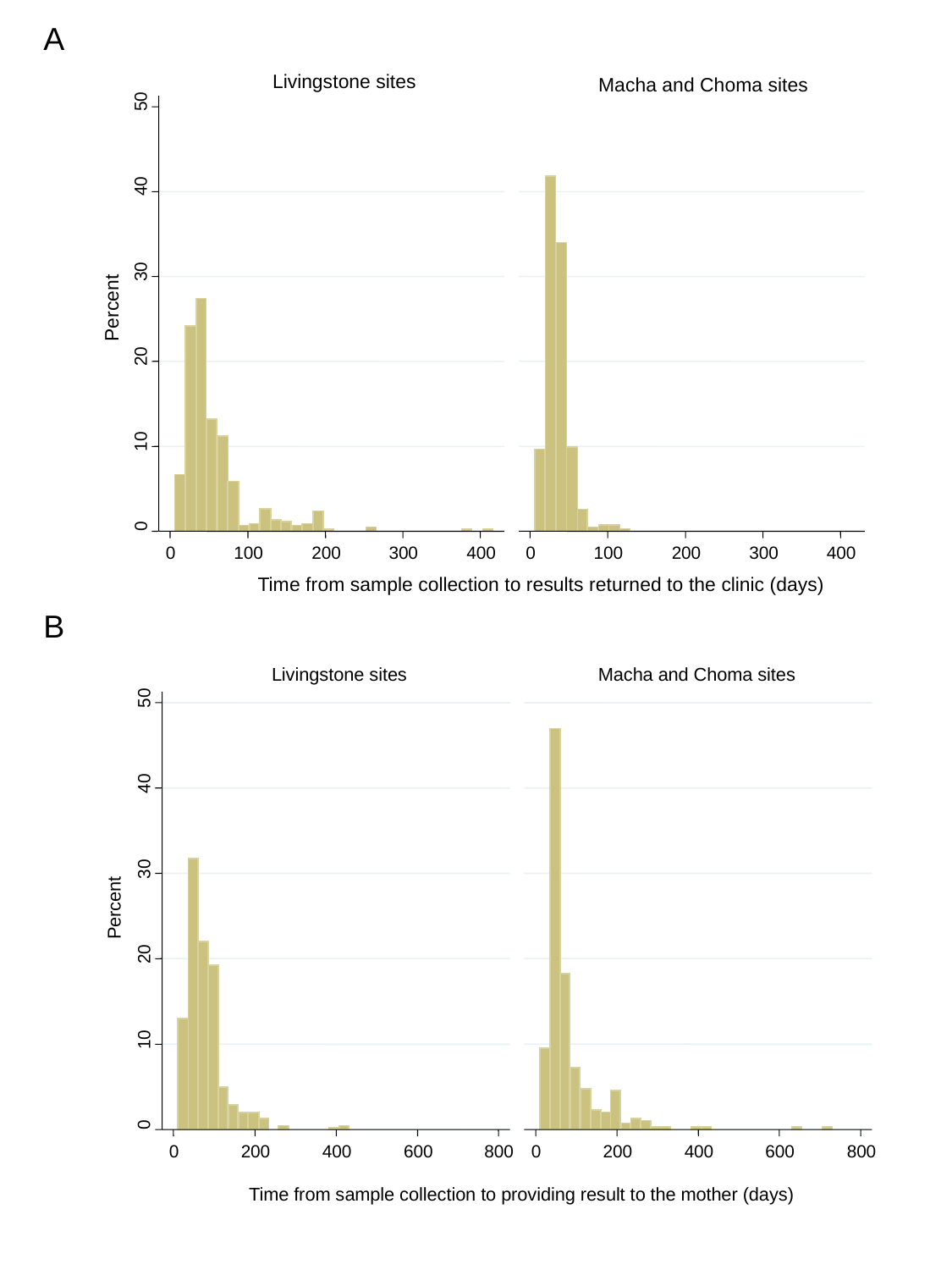

A
Livingstone sites
Macha and Choma sites
50
40
30
Percent
20
10
0
0
100
200
300
400
0
100
200
300
400
Time from sample collection to results returned to the clinic (days)
B
Livingstone sites
Macha and Choma sites
50
40
30
Percent
20
10
0
0
200
400
600
800
0
200
400
600
800
Time from sample collection to providing result to the mother (days)
