# Supplementary material for: Acceptability and feasibility of testing for HIV infection at birth and linkage to care in rural and urban Zambia: a cross-sectional study
Source: BMC Infect Dis. 2020 Mar 18;20:227. doi: 10.1186/s12879-020-4947-6 (PMC7079396; doi:10.1186/s12879-020-4947-6)
Supplement: Supplementary file 5 — Additional file 5. Median turnaround time (in days) for test results in Livingstone by operational period. Period 1: sample collected June 30, 2016 (study start) to September 30, 2016 – normal lab operation with one PCR instrument; Period 2: sample collected October 1, 2016 to December 13, 2016 – reagent stockout; Period 3: sample collected December 14, 2016 to August 17, 2017 – normal lab operation with one PCR instrument; Period 4: sample collected August 18, 2017 to October 30, 2017 – normal lab operation with two PCR instruments; Period 5: sample collected October 31, 2017 to December 14, 2017 – reagent stockout; Period 6: sample collected December 15, 2017 to April 10, 2018 (study end) – normal lab operation with two PCR instruments. [file 12879_2020_4947_MOESM5_ESM.pptx]

## Slide 1
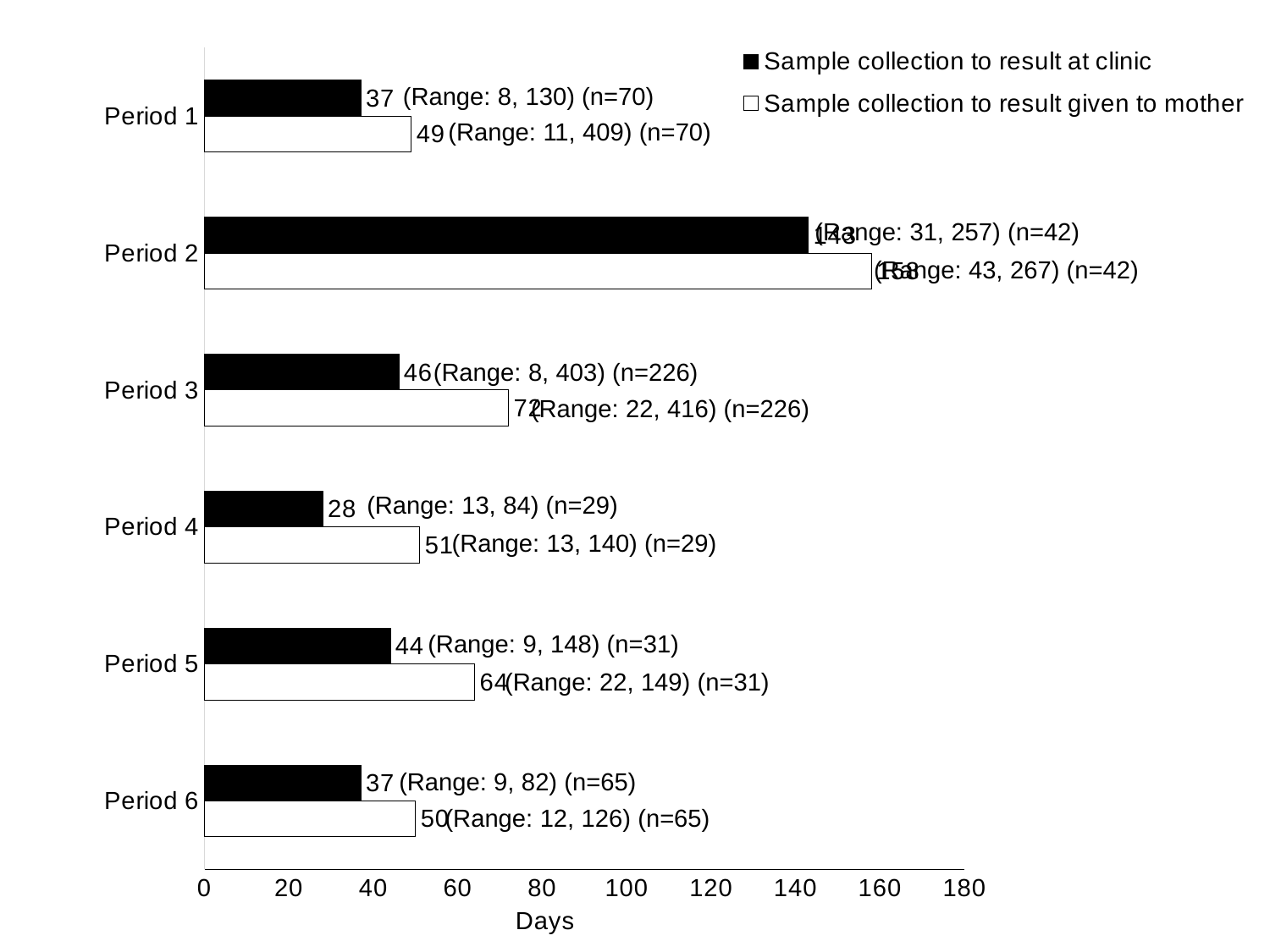

### Chart
| Category | Sample collection to result given to mother | Sample collection to result at clinic |
|---|---|---|
| Period 6 | 50.0 | 37.0 |
| Period 5 | 64.0 | 44.0 |
| Period 4 | 51.0 | 28.0 |
| Period 3 | 72.0 | 46.0 |
| Period 2 | 158.0 | 143.0 |
| Period 1 | 49.0 | 37.0 |(Range: 8, 130) (n=70)
(Range: 11, 409) (n=70)
(Range: 31, 257) (n=42)
(Range: 43, 267) (n=42)
(Range: 8, 403) (n=226)
(Range: 22, 416) (n=226)
(Range: 13, 84) (n=29)
(Range: 13, 140) (n=29)
(Range: 9, 148) (n=31)
(Range: 22, 149) (n=31)
(Range: 9, 82) (n=65)
(Range: 12, 126) (n=65)
