# Supplementary material for: Acceptability and feasibility of testing for HIV infection at birth and linkage to care in rural and urban Zambia: a cross-sectional study
Source: BMC Infect Dis. 2020 Mar 18;20:227. doi: 10.1186/s12879-020-4947-6 (PMC7079396; doi:10.1186/s12879-020-4947-6)
Supplement: Supplementary file 7 — Additional file 7. Reasons that HIV test results were not returned to mothers by study location [file 12879_2020_4947_MOESM7_ESM.docx]

**Additional File 7. Reasons that HIV test results were not returned to mothers by study location**

|  | **Livingstone City** | | **Choma Town** | | **Macha** | |
| --- | --- | --- | --- | --- | --- | --- |
|  | **Hospital**  **N=485** | **Urban health centers**  **N=305** | **Hospital**  **N=63** | **Urban health center**  **N=38** | **Hospital**  **N=190** | **Rural health centers**  **N=209** |
| Did not receive test results, n (%) | 235 (48.5) | 92 (30.2) | 9 (14.3) | 3 (7.9) | 58 (30.5) | 30 (14.4) |
| Reason, n (%) |  |  |  |  |  |  |
| Defaulted | 116 (49.4) | 10 (10.9) | 0 | 0 | 21 (36.2) | 21 (70.0) |
| No phone | 71 (30.2) | 45 (48.9) | 1 (11.1) | 1 (33.3) | 25 (43.1) | 0 |
| Not reachable | 44 (18.7) | 23 (25.0) | 4 (44.4) | 0 | 10 (17.2) | 0 |
| Wrong number given | 3 (1.3) | 4 (4.4) | 3 (33.3) | 0 | 0 | 0 |
| Mother/child moved | 0 | 5 (5.4) | 1 (11.1) | 2 (66.7) | 1 (1.7) | 3 (10.0) |
| Staff oversight | 0 | 0 | 0 | 0 | 1 (1.7) | 6 (20.0) |
| Child died | 1 (0.4) | 3 (3.3) | 0 | 0 | 0 | 0 |
| DBS lost | 0 | 2 (2.2) | 0 | 0 | 0 | 0 |
