# Supplementary material for: Acceptability and feasibility of testing for HIV infection at birth and linkage to care in rural and urban Zambia: a cross-sectional study
Source: BMC Infect Dis. 2020 Mar 18;20:227. doi: 10.1186/s12879-020-4947-6 (PMC7079396; doi:10.1186/s12879-020-4947-6)
Supplement: Supplementary file 8 — Additional file 8. Factors associated with receiving test results for early infant diagnosis by study location [file 12879_2020_4947_MOESM8_ESM.docx]

**Additional File 8. Factors associated with receiving test results for early infant diagnosis by study location**

|  | **Livingstone City**  **N=790** | | **Choma Town**  **N=101** | | **Macha**  **N=398** | |
| --- | --- | --- | --- | --- | --- | --- |
|  | **Received results**  **n / N (%)** | **p-value** | **Received results**  **n / N (%)** | **p-value** | **Received results**  **n / N (%)** | **p-value** |
| Type of facility |  | <0.0001 |  | 0.34 |  | <0.0001 |
| Hospital | 250 / 485 (51.6) |  | 54 / 63 (85.7) |  | 132 / 190 (69.5) |  |
| Urban/rural health center | 213 / 305 (69.8) |  | 35 / 38 (92.1) |  | 179 / 209 (85.7) |  |
| Returning to study location for post-natal care |  | 0.008 |  | 0.13 |  | <0.0001 |
| No | 336 / 600 (56.0) |  | 54 / 64 (84.4) |  | 82 / 138 (59.4) |  |
| Yes | 127 / 190 (66.8) |  | 35 / 37 (94.6) |  | 229 (261 (87.7) |  |
| Transport time to the facility (hours)† |  | 0.12 |  | 0.44 |  | 0.02 |
| Short | 376 / 635 (59.2) |  | 56 / 62 (90.3) |  | 109 / 131 (83.2) |  |
| Medium | 55 / 94 (58.5) |  | 12 / 16 (75.0) |  | 84 / 99 (84.9) |  |
| Long | 10 / 22 (45.5) |  | 6 / 7 (85.7) |  | 57 / 78 (73.1) |  |
| Very long | 4 / 13 (30.8) |  | 8 / 9 (88.9) |  | 51 / 74 (68.9) |  |
| Mother’s age (years) |  | 0.06 |  | 0.88 |  | 0.61 |
| <30 | 233 / 420 (55.5) |  | 54 / 61 (88.5) |  | 150 / 190 (79.0) |  |
| ≥30 | 230 / 370 (62.2) |  | 35 / 40 (87.5) |  | 159 / 207 (76.8) |  |
| Mother has access to a cell phone |  | <0.0001 |  | 0.58 |  | 0.29 |
| No | 54 / 183 (29.5) |  | 23 / 27 (85.2) |  | 111 / 148 (75.0) |  |
| Yes | 409 / 607 (67.4) |  | 66 / 74 (89.2) |  | 199 / 250 (79.6) |  |
| Mother’s education |  | <0.0001 |  | 0.53 |  | 0.63 |
| None/Primary | 87 / 182 (47.8) |  | 34 / 37 (91.9) |  | 175 / 229 (76.4) |  |
| Secondary | 305 / 517 (59.0) |  | 50 / 58 (86.2) |  | 123 / 153 (80.4) |  |
| More than secondary | 68 / 88 (77.3) |  | 4 / 4 (100.0) |  | 9 / 11 (81.8) |  |
| Father’s education |  | <0.0001 |  | 0.02 |  | 0.38 |
| None/Primary | 17 / 32 (53.1) |  | 12 / 17 (70.6) |  | 97 / 130 (74.6) |  |
| Secondary | 268 / 502 (53.4) |  | 61 / 65 (93.9) |  | 142 / 175 (81.1) |  |
| More than secondary | 124 / 160 (77.5) |  | 9 / 10 (90.0) |  | 16 / 21 (76.2) |  |
| High-risk infant‡ |  | 0.49 |  | --- |  | 0.002 |
| No | 341 / 589 (57.9) |  | 0 / 0 |  | 212 / 256 (82.8) |  |
| Yes | 122 / 201 (60.7) |  | 12 / 89 (88.1) |  | 98 / 142 (69.0) |  |

† Livingstone and Choma: Short=<30 minutes, medium=30-59 minutes, long=1.0-1.9 hours, very long=≥2.0 hours; Macha: short=<1 hour, medium=1.0-1.9 hours, long=2.0-2.9 hours, very long==≥3.0 hours

‡ High-risk was defined as not receiving antiretroviral drugs throughout pregnancy or starting antiretroviral drugs during pregnancy to prevent mother-to-child transmission of HIV
