# Supplementary material for: Acceptability and feasibility of testing for HIV infection at birth and linkage to care in rural and urban Zambia: a cross-sectional study
Source: BMC Infect Dis. 2020 Mar 18;20:227. doi: 10.1186/s12879-020-4947-6 (PMC7079396; doi:10.1186/s12879-020-4947-6)
Supplement: Supplementary file 9 — Additional file 9. Timing of testing and ART initiation for HIV-infected infants linked to care [file 12879_2020_4947_MOESM9_ESM.docx]

**Additional File 9. Timing of testing and ART initiation for HIV-infected infants linked to care**

|  | **Study area** | **Days from sample collection to result at the clinic** | **Days from result at the clinic to result given to the mother** | **Days from result given to the mother to ART initiation** | **Days from sample collection to ART initiation** | **Comment** |
| --- | --- | --- | --- | --- | --- | --- |
| **1** | Livingstone | 26 | 5 | 0 | 31 |  |
| **2** | Livingstone | 57 | 3 | 11 | 71 |  |
| **3** | Livingstone | 36 | 0 | 0 | 36 |  |
| **4** | Choma | 35 | 112 | 13 | 160 | Mother did not return for the results and was traced after 3.5 months |
| **5** | Choma | 56 | 0 | 8 | 64 |  |
| **6** | Macha | 28 | 1 | 0 | 29 |  |
| **7** | Macha | 28 | 34 | 123 | 185 | Mother referred to health facility outside of study area |
| **8** | Macha | 39 | 30 | 715 | 784 | Mother defaulted for a year after receiving the results. After being traced by clinic staff, the child had a negative rapid test and the accompanying dried blood spot card was lost. After being traced a second time and re-tested, the child had a positive HIV DNA result and was linked to care. |
