# Supplementary material for: Acceptability and feasibility of testing for HIV infection at birth and linkage to care in rural and urban Zambia: a cross-sectional study
Source: BMC Infect Dis. 2020 Mar 18;20:227. doi: 10.1186/s12879-020-4947-6 (PMC7079396; doi:10.1186/s12879-020-4947-6)
Supplement: Supplementary file 10 — Additional file 10. Characteristics of women living with HIV and their infants attending study sites for delivery and post-natal care, 2014–2017 [file 12879_2020_4947_MOESM10_ESM.docx]

**Additional File 10. Characteristics of women living with HIV and their infants attending study sites for delivery and post-natal care, 2014-2017**

|  | **Livingstone City** | | | **Choma Town** |  |
| --- | --- | --- | --- | --- | --- |
|  | **Mahatma Gandhi Clinic**  **(n=303)** | **Libuyu Clinic**  **(n=429)** | **Maramba Clinic**  **(n=1031)** | **Shampande Clinic**  **(n=315)** | **Overall**  **(n=2078)** |
| Maternal age, median (IQR) | 27 (24, 32) | 29 (23, 33) | 28 (24, 32) | 28 (24, 33) | 28 (24, 32) |
| Place of delivery, n (%) |  |  |  |  |  |
| Facility | 289 (95.4) | 393 (91.6) | 981 (95.2) | 181 (57.5) | 1844 (88.7) |
| Hospital | 2 (0.7) | 5 (1.2) | 3 (0.3) | 0 | 10 (0.5) |
| Home | 0 | 3 (0.7) | 4 (0.4) | 1 (0.3) | 8 (0.4) |
| Born before arrival | 7 (2.3) | 16 (3.7) | 30 (2.9) | 0 | 53 (2.6) |
| Unknown | 5 (1.7) | 12 (2.8) | 13 (1.3) | 133 (42.2) | 163 (7.8) |
| Origin, n (%) |  |  |  |  |  |
| Within catchment area (<12 km) | 288 (95.1) | 405 (94.4) | 921 (89.3) | 231 (73.3) | 1845 (88.8) |
| Within catchment area (>12 km) | 11 (3.6) | 5 (1.2) | 58 (5.6) | 15 (4.8) | 89 (4.3) |
| Outside of catchment area | 1 (0.3) | 7 (1.6) | 20 (1.9) | 19 (6.0) | 47 (2.2) |
| Unknown | 3 (1.0) | 12 (2.8) | 32 (3.1) | 50 (15.9) | 97 (4.7) |
| Mode of delivery, n (%) |  |  |  |  |  |
| Vaginal delivery | 298 (98.4) | 396 (92.3) | 1001 (97.1) | 259 (82.2) | 1954 (94.0) |
| Caesarian section | 2 (0.7) | 19 (4.4) | 17 (1.7) | 8 (2.5) | 46 (2.2) |
| Unknown | 3 (1.0) | 14 (3.3) | 13 (1.3) | 48 (15.2) | 78 (3.8) |
| Baby sex, n (%) |  |  |  |  |  |
| Male | 124 (40.9) | 138 (32.2) | 419 (40.6) | 118 (37.5) | 799 (38.5) |
| Female | 144 (47.5) | 165 (38.5) | 426 (41.3) | 82 (26.0) | 817 (39.3) |
| Unknown | 35 (11.6) | 126 (29.4) | 186 (18.0) | 115 (36.5) | 462 (22.2) |
| Condition of baby, n (%) |  |  |  |  |  |
| Live birth | 298 (98.4) | 412 (96.0) | 1010 (98.0) | 265 (84.1) | 1985 (95.5) |
| Still birth | 1 (0.3) | 4 (0.9) | 6 (0.6) | 1 (0.3) | 12 (0.6) |
| Unknown | 4 (1.3) | 13 (3.0) | 15 (1.5) | 49 (15.6) | 81 (3.9) |
